# Supplementary material for: Sound source localization for spatial mapping of surgical actions in dynamic scenes
Source: Int J Comput Assist Radiol Surg. 2026 Jul 3;21(7):1603–11. doi: 10.1007/s11548-026-03745-6 (PMC13553653; doi:10.1007/s11548-026-03745-6)
Supplement: Supplementary file 1 [file 11548_2026_3745_MOESM2_ESM.pdf]

Sound Source Localization for Spatial Mapping of Surgical Actions in Dynamic Scenes

Supplementary Material

Jonas Hein[1,3], Lazaros Vlachopoulos[2], Maurits Geert Laurent Olthof[2], Bastian Sigrist[1], Philipp Fürnstahl[1], Matthias Seibold[1*]

[1]ROCS, Balgrist University Hospital, University of Zurich, Switzerland.
[2]Dept. of Orthopedics, Balgrist University Hospital, University of Zurich, Switzerland.
[3]Computer Vision and Geometry Group, ETH Zurich, Switzerland.

*Corresponding author(s). E-mail(s): matthias.seibold@balgrist.ch;

This supplementary material provides technicalities and additional results of the experiments that were omitted in the main paper due to space limitations. In Table 1 we report the accuracy of our proposed event localization stage under a range of IoU thresholds from 0.05 to 0.4 . Figure 2 shows representative failure cases of our proposed pipeline.

Ablation: OR Noise

We performed a simple evaluation of the robustness of the event detection stage under simulated realistic OR background noise, by adding OR background noise from a publicly available recording[1] to the test set and reporting the result in Table 2.

Ablation: Naive Centroid Detection Baseline

Figure 1 displays the error distribution of the naive centroid detection baseline, which computes the 3D sound source location as the weighted average of the entire point cloud, using the sound amplitude as per-point weights.

[1]https://freesound.org/people/nicotep/sounds/632733/, License: Creative Commons 0

Table 1: Recall rates of our proposed event localization stage. A successful event localization is defined by the 3D bounding box IoU above a defined threshold.

IoU Threshold	Chiseling	Drilling	Sawing	Average
0.05	0.81	0.94	0.91	0.88
0.10	0.78	0.91	0.84	0.84
0.20	0.69	0.47	0.42	0.52
0.40	0.34	0.00	0.00	0.12

Table 2: Results with realistic OR noise added to the test set

Method	Precision [%]		Recall [%]		F1 [%]	
	**Hard**	**Relaxed**	**Hard**	**Relaxed**	**Hard**	**Relaxed**
Chiseling	0.909 ± 0.058	**0.975 ± 0.029**	0.763 ± 0.152	**0.940 ± 0.057**	0.825 ± 0.110	**0.957 ± 0.042**
Drilling	0.000 ± 0.000	**0.711 ± 0.092**	0.000 ± 0.000	**0.663 ± 0.174**	0.000 ± 0.000	**0.659 ± 0.075**
Sawing	0.444 ± 0.076	**1.000 ± 0.000**	0.389 ± 0.079	**0.889 ± 0.157**	0.411 ± 0.069	**0.933 ± 0.094**

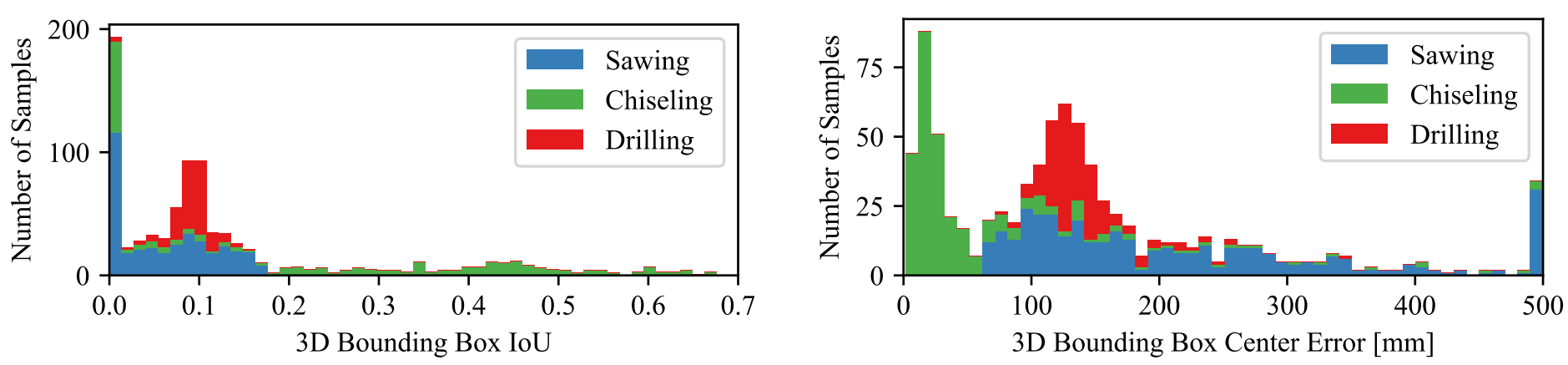

Figure 1: Distribution of 3D bounding box IoU scores and 3D bounding box center errors of the naive centroid detection baseline, over a total of 20 recordings, grouped by surgical action.

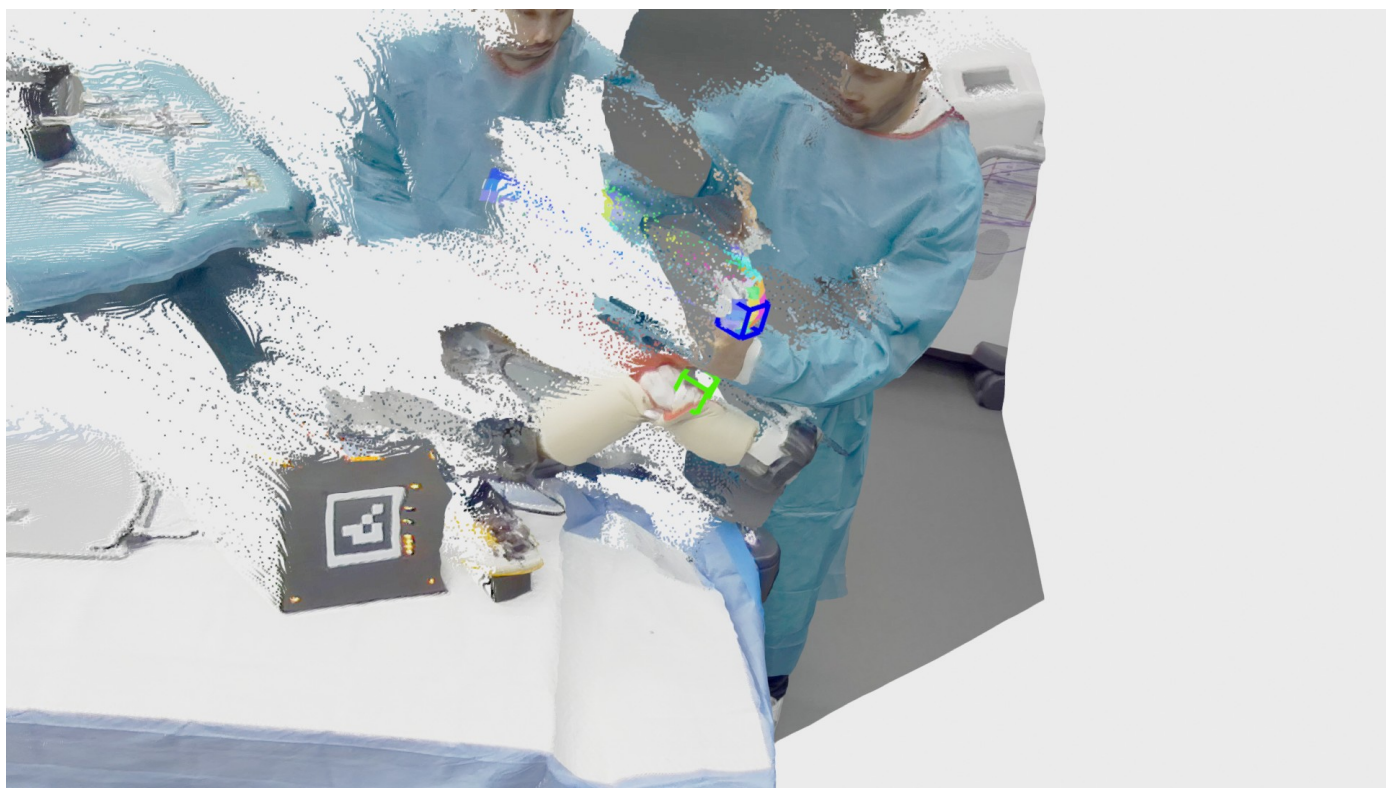

(a) Acoustic Artifacts

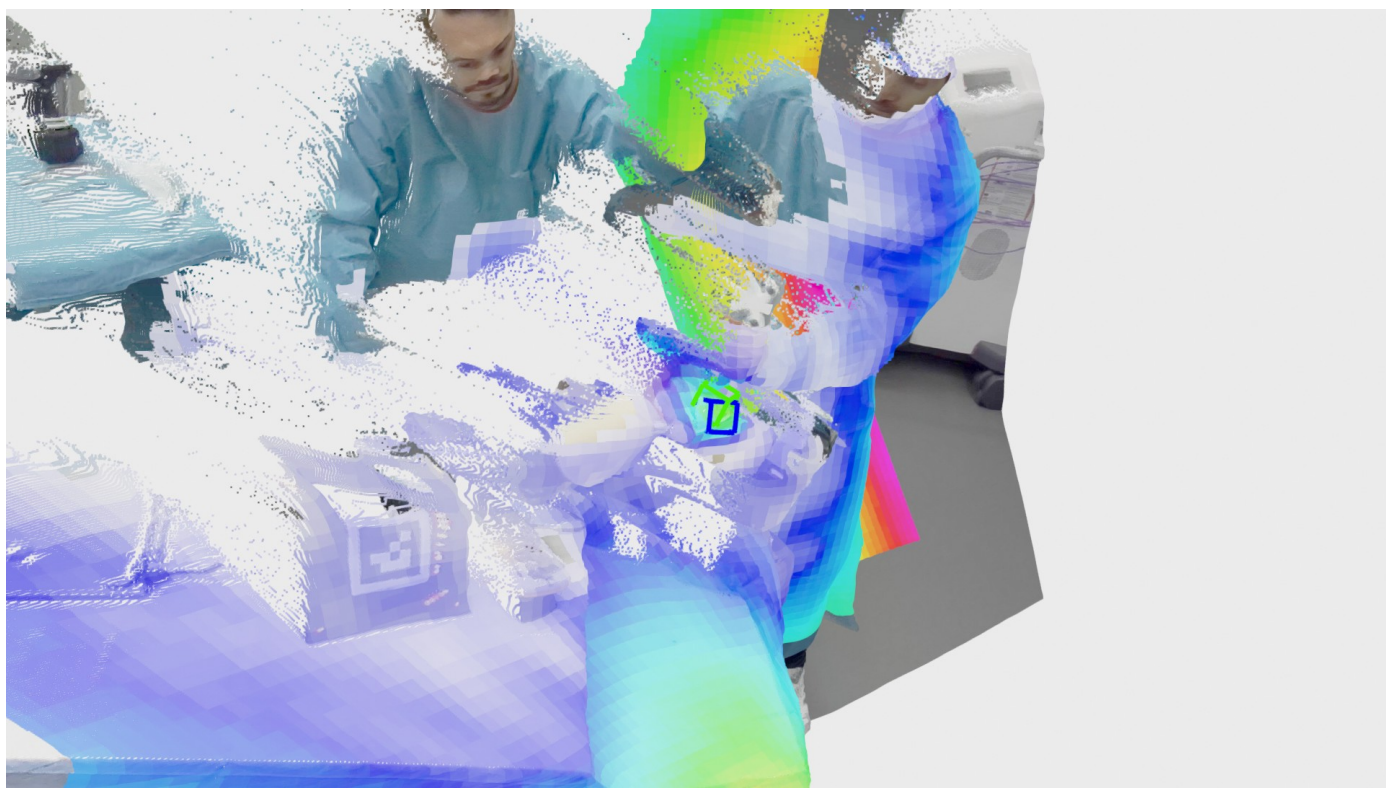

(b) Acoustic Reflections

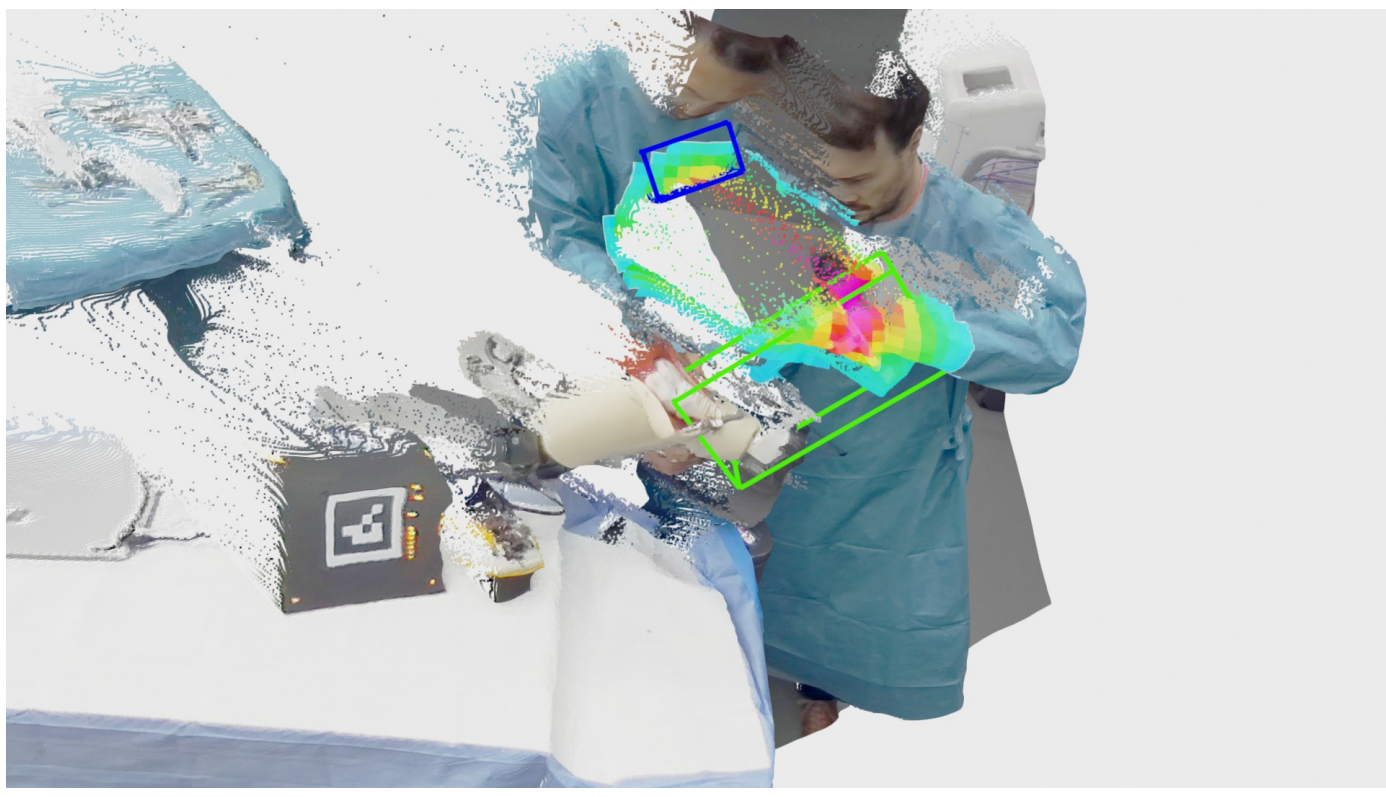

(c) Incorrect Cluster

Figure 2: Failure cases for the proposed pipeline.
